# The Impact of Music on Stress Reduction and Academic Performance of Dental Students

**DOI:** 10.7759/cureus.46554

**Published:** 2023-10-05

**Authors:** Ubaydah F Algailani, Bereket M Tigabu, Yad R Rahim, Ahmed A Alzbeede, Lubna O Alshaikhli

**Affiliations:** 1 Department of Dentistry, College of Medicine, Komar University of Science and Technology, Sulaymaniyah, IRQ; 2 Department of Pharmacy, College of Medicine, Komar University of Science and Technology, Sulaymaniyah, IRQ; 3 Department of Medical Laboratory Science, College of Science, Komar University of Science and Technology, Sulaymaniyah, IRQ; 4 Department of Periodontics, College of Dentistry, University of Sulaimani, Sulaymaniyah, IRQ

**Keywords:** kurdistan region of iraq, undergraduate dental students, preclinical dental students, stress reduction, perceived stress scale, music therapy, dental education, academic performance

## Abstract

Background

Dental education, one of the most demanding healthcare fields, is linked to higher physical and mental illnesses in students. Stress, which arises when demands surpass an individual's ability to adapt, can lead to disruptions in cognition, mood, and behavior. Due to the intense academic pressure, dental students are susceptible to stress and other mental issues. Music therapy, an ancient practice, is now popular for stress reduction. However, its effect on academic performance is debated.

Objectives

This study aimed to evaluate the impact of music on stress levels and academic performance of dental students.

Methods

The study included third-year dental students from the College of Dentistry, Komar University of Science and Technology, Sulaymaniyah, Iraq. The demographic data were collected using a self-administered questionnaire.

Results

The study found that music intervention reduced the perceived stress levels of dental students in the intervention group compared to the baseline but did not reach a significant level. The final stress levels were positively correlated with baseline stress levels but did not correlate with other parameters. No association between sociodemographic variables and the Perceived Stress Scale was found. The study also found no significant positive or negative effect of music on academic performance.

Conclusion

The study suggests that music may positively impact stress reduction for dental students during their academic years in dental school. It highlighted the significance of stress-reduction programs in the academic curriculum in lowering dentistry students' stress and, as a result, potentially improving their learning abilities.

## Introduction

Dental education involves a comprehensive and rigorous curriculum that prepares students for the practice of dentistry. Dental students are expected to have a solid scientific foundation and must also demonstrate excellent manual dexterity to perform the delicate procedures required in dentistry. The dental practice has long been regarded as one of the most demanding healthcare education fields. Dental students appear to be experiencing higher physical and mental illnesses in general practice than students from other scientific backgrounds. This has been linked to work stress in preclinical dentistry [1].

Stress is a condition that occurs when surrounding demands exceed an individual's adaptive capability, resulting in psychological or biological changes. Immediate stress can cause transient disruptions in cognition, mood, and behavior, harming the person's well-being [2]. Because of the intense academic pressure, dental students are more prone to developing stress and other mental issues and a diverse environment. According to reports, 15% to 25% of medical students exhibit various behaviors and psychological issues throughout their studies in medical college [3]. Multiple factors contribute to those students' mental illness and health problems, which affect their careers [4].

Students transferring from high school or college to a professional institute undergo a major life adjustment. The initial years of dental school are the most difficult in a student's life due to the unfamiliar environment, unusual teaching and learning methods, increasing academic demand, fear of failure, and heavy workload [5]. Several studies have indicated that dental students experience personal discomfort often during their undergraduate education. Chronic stress can result in anxiety and despondency [6]. Besides, dental students face numerous challenges daily in the workplace, such as working in uncomfortable positions, high noise levels, defective equipment, financial issues, staff coordination, time and scheduling pressures, administrative responsibilities, extended periods of concentration, and social isolation [7].

Dental college programs are distinct from other academic programs due to their inclusion of comprehensive pre-clinical and clinical courses, which necessitate the acquisition of a diverse range of skills, including psychomotor abilities, interpersonal aptitude, responsibility, and effective communication capabilities. Hence, the incorporation of non-cognitive factors may prove valuable in effectively forecasting the academic achievement of dental students [8].

Music therapy is becoming popular as a stimulator to reduce stress. Sound in treating diseases dates back to ancient times, with Egyptian, Greek, Chinese, Indian, and Roman inscriptions extolling music as healing [9]. Music therapy is a low-cost, non-invasive method of reducing students’ stress. So far, various research studies have been conducted in which the influence of music on stress reduction has been explored in diverse groups [9]. In addition, background music (BM) might be seen as a stimulant that enhances students' academic performance. While for some, it may be a distracting source of noise that hinders their productivity at work [10].

This study intends to assess the influence of music on the stress of dentistry school students and its relationship to their pre-clinical laboratory performance.

## Materials and methods

Third-year dental students were selected from the College of Dentistry, Komar University of Science and Technology, Sulaymaniyah, Iraq. The demographic data were collected using a self-administered questionnaire technique. Before data collection, students were briefed on the study objectives, a study information sheet was provided, and written consent from study participants was obtained. All information gathered was kept private.

The students were separated into two groups; the baseline information was collected a week before the initiation of the intervention. The academic performance and the level of stress were evaluated at three different procedures in three different weeks. The average of the three sessions was taken as the level of academic performance and stress. The control group made cavity preparations according to the GV Black principle in two hours without BM, whereas the intervention group performed the same procedure with BM. In the intervention group, the music played on a sound system during the study, which was picked beforehand, was classical music of Mozart, Beethoven, Bach, Chopin, and Vivaldi. Participants’ works were evaluated accordingly by the same evaluator. The entire evaluation was done in a double-blind process, and all students were trained in the same laboratory procedures before the beginning of the study, and participation in the training sessions was mandatory. The entire study was done in the phantom lab and involved cavity preparation on the phantom model.

The stress level of the participants was evaluated during the study using the Perceived Stress Scale (PSS) test, which was conducted during the lab procedure to avoid bias. The PSS is a widely used psychological instrument to measure the perception of stress, in which the subjects must answer 10 questions (items) related to feelings and thoughts experienced as stress during the lab session. The score for each item ranges from 0 to 4, where 0 = never, 1 = almost never, 2 = sometimes, 3 = fairly often, and 4 = very often. The total score ranges from 0 to 40. The subjects with high PSS scores are considered to have stress. The responses were summed up so that higher scores indicated more perceived stress.

A pilot study was carried out on a small sample of students to test the validity of the self-developed questionnaire and needed amendments to be made. Cronbach's alpha was 0.79, which can be considered a good indication of the internal consistency of the questions presented in our questionnaire. Points were calculated according to the calculation system of the scale proposed by Cohen et al. [11].

The study was approved by the Research Ethics Committee of Komar University of Science and Technology (approval number: S22-02-DEN-01). The statistical analysis was performed using STATA 14 statistical software (StataCorp LLC, College Station, TX), and a P-value less than 0.05 was taken as a cut-off for significance.

## Results

Demographic information of the subjects is presented in Table 1. A total of 47 students were included in the current study: 22 males and 25 females. The mean age of students was 21 ± 1.1 years. Of the students, 27 were allocated to the control group and 20 students were included in the intervention group. There was no statistically significant difference between the intervention and the control groups regarding the baseline criteria. Skewness and kurtosis tests were checked for the normality of distribution and the values were less than +1 for both, which showed that the data were normally distributed.

**Table 1:** Baseline characteristics of study participants. cGPA: cumulative grade point average.

		Control (n = 27)	Intervention (n = 20)	P-value
Age (average ± SD)				
		21.1 ± 1.1	20.9 ± 1.1	0.592
Gender				
	Female	17 (63.0%)	8 (40.0%)	0.119
	Male	10 (37.0%)	12 (60.0%)	
cGPA (average ± SD)				
		2.9 ± 0.35	2.9 ± 0.31	0.892
Family status				
	Living together	26 (96.3%)	16 (80.0)	0.194
	Separated	0	1 (5.0%)	
	Recently lost either one of the family	1 (3.7%)	3 (15.0%)	
Residential area				
	Sulaymaniyah	25 (92.6%)	16 (80.0%)	0.379
	Out of Sulaymaniyah	2 (7.4%)	4 (20.0%)	
Family size (average ± SD)				
		5.3 ± 0.91	4.9 ± 1.3	0.183
Lost family member				
	Yes	2 (7.4%)	1 (5.0%)	1.000
	No	25 (92.6%)	19 (95.0%)	
Extra-hour work				
	Yes	0	3 (15.0%)	0.070
	No	27 (100.0%)	17 (85.0%)	
Baseline academic performance				
		8.2 ± 0.88	7.85 ± 1.13	0.233
Baseline perceived stress				
		14.4 ± 7.64	14.9 ± 5.25	0.825
Baseline perceived stress category				
	Low stress	14 (51.9%)	8 (40.0%)	0.365
	Moderate stress	11 (40.7%)	12 (60.0%)	
	High stress	2 (7.4%)	0	

**Figure 1:**
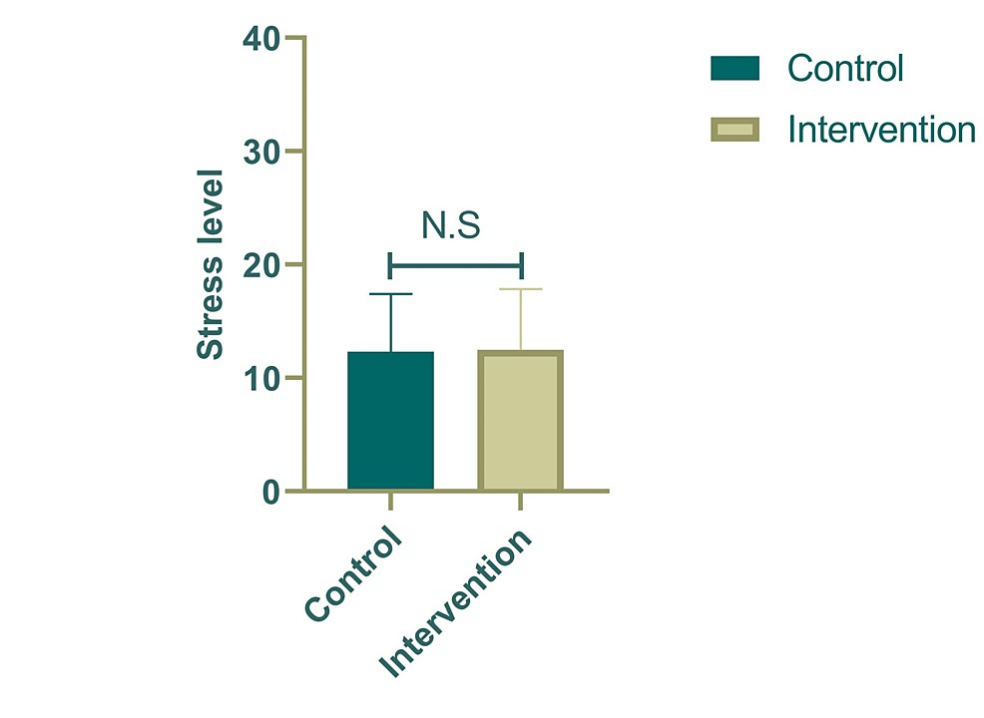
Effect of music on perceived stress in both control and intervention groups.

**Figure 2:**
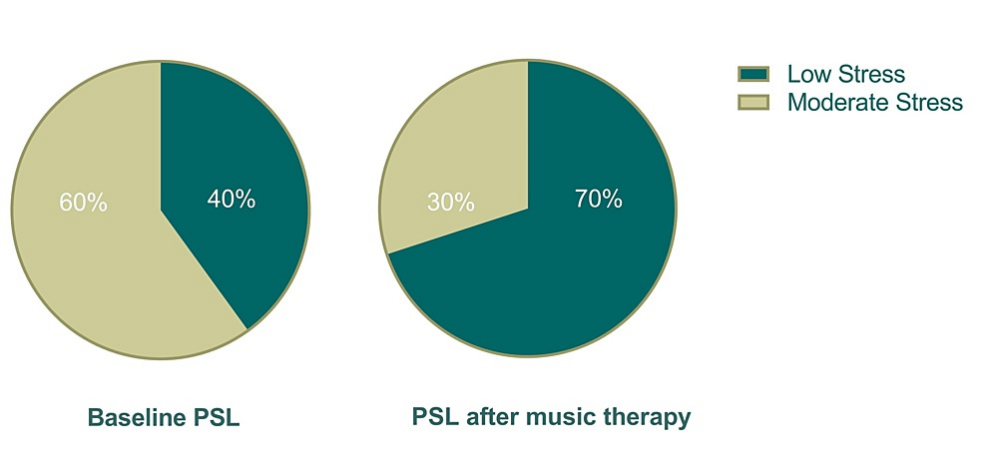
Perceived stress level in the intervention group before and after using the music background. PSL: perceived stress level.

In the present study, music intervention had no statistically significant reduction in the perceived stress of students (12.48 ± 5.36 vs. 12.31 ± 5.11; P = 0.910) (Figure 1). The final stress was positively correlated with baseline stress (P = 0.003) rather than the other parameters (Table 2). None of the sociodemographic variables were statistically associated with the PSS (Table 2). Nevertheless, the number of students with moderate stress declined by 50% in the intervention group and none of the students had severe stress (Figure 2).

**Table 2:** Pairwise correlation of stress level and academic performance. cGPA: cumulative grade point average. * Statistically significant.

	Age	cGPA	Baseline academic performance	Baseline stress level	Final academic performance	Final stress level
Age	1.000					
cGPA	-0.109 (0.466)	1.000				
Baseline academic performance	-0.181 (0.223)	0.209 (0.158)	1.000			
Baseline stress level	0.334 (0.022*)	0.191 (0.199)	-0.018 (0.905)	1.000		
Final academic performance	-0.172 (0.249)	-0.100 (0.504)	0.084 (0.573)	-0.074 (0.622)	1.000	
Final stress level	0.182 (0.222)	-0.067 (0.656)	-0.074 (0.622)	0.423 (0.003*)	-0.052 (0.731)	1.000

**Figure 3:**
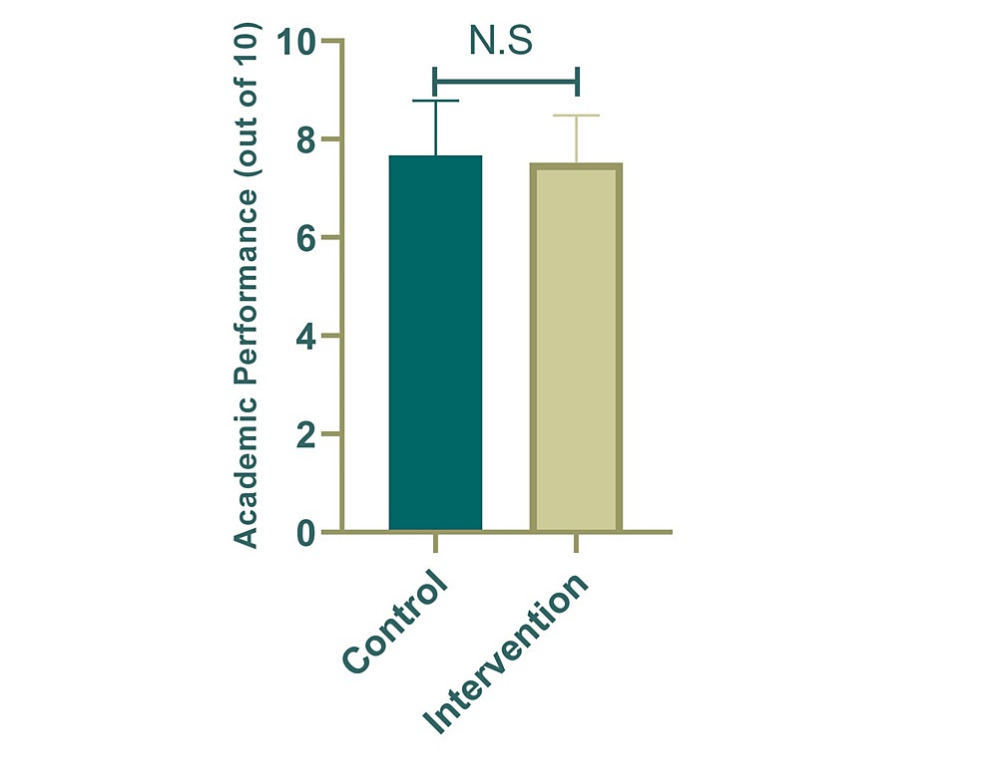
The effect of music on academic performance.

Regarding academic performance, the music background does not seem to affect the student's academic performance either negatively or positively (7.52 ± 0.97 vs. 7.67 ± 1.12; P = 0.620) (Figure 3).

## Discussion

This study aims to evaluate the impact of music on the stress levels of dental school students and how it relates to their performance in pre-clinical laboratories. The study found that music intervention did not have a statistically significant reduction in the perceived stress levels of dental students. The final stress levels were positively correlated with baseline stress levels but did not correlate with other parameters. No association between sociodemographic variables and the PSS was found.

However, there was a 50% reduction in moderate stress levels in the intervention group, and no students in this group had severe stress. The study also found no significant effect of music on academic performance. The notion that attending dental school is stressful for students is growing in popularity among educators. Both Western and Asian dental students experience significant levels of stress [12-16].

Academic issues are the top stressors perceived by dental students, which is consistent with previous research [17,18]. Fear of failure in a course was ranked as the most stressful item across all professional years, which appeared to be consistent with Tangade and colleagues' findings [18]. These findings may help improve strategies that may enable students to overcome academic life-related stressors.

It was shown that the majority of dental students find preclinical laboratories to be quite difficult. Not all students are adept at transitioning from the fundamental science of the first two years of dental school to preclinical dentistry in the third year. Sadly, Iraq has not yet seen the results of such research. This study, which examines the stress experienced by undergraduate dentistry students, may be the first of its kind. Due to the difference in stress levels between preclinical and clinical work on patients, fifth and fourth-year dental students were excluded from the research [19].

This investigation aimed to evaluate the effect of BM in reducing stress during preclinical dental laboratories and its impact on academic performance. Our study confirmed the general impression that dental students experience significant stress and anxiety, which is consistent with previous research [20].

The PSS was employed in the current investigation to assess the stress levels of each participant. According to recent research, a PSS score greater than 20 indicates significant stress [21]. Academic factors were the predominant stressors for students. Multiple examinations and daily assessments in the laboratory with the limitation of the training time to accomplish the clinical practice all contribute to the stress that students face, along with living away from home and tuition fee concerns [15].

The intervention group showed a 50% reduction in stress levels after using BM; this can be attributed to music, which was able to produce a less stressful environment, time utilization, attentiveness, and enhance students' interest, as evidenced by the overwhelming response from students recommending playing the BM in the laboratories. However, it was not statistically significant. This is supported by a study done on Iranian dental students, which showed that BM can reduce the anxiety and depression of dental students [20,22]. Multiple reports on using BM claim that it alters mood, shifts temporal orientation, triggers physiological changes, minimizes anxiety, calms the mind, lowers distraction, enhances focus, and supports performance [23-25].

On the other side, research revealed that music may distract students, which might have an impact on how well they do academically [26]. Also, Cassidy and MacDonald assert that compared to quiet, the students' performance suffered in the presence of BM [27]. This current study illustrated that students were not affected negatively by the music background, which shows a good level of concentration during the lab session. Furthermore, BM did not improve their academic performance. This could be due to music having specific effects relating to processing requirements, e.g., form, complexity, genre, familiarity, and tempo [27].

Limitations of the study

The study's scope is restricted to a single institution and a specific year of study, potentially limiting the generalizability of the results. Additionally, the impact of different types of music, individual musical preferences, or varying volumes was not assessed, which could influence the outcome. Lastly, the study emphasizes perceived stress levels using the PSS and does not incorporate other physiological measures of stress or comprehensive psychological evaluations. Future studies might delve deeper into these aspects to offer a more holistic view of stress and its management in dental education.

## Conclusions

The results of this study suggest that playing music during dentistry students' laboratory sessions might help alleviate their tension without negatively impacting their academic performance. Our findings once again underline the need to include stress-reduction programs in the dental school curriculum to reduce stress levels among dental students, which may therefore improve their academic performance.
